# Effect of Graphene Oxide (GO) on the Morphology and Microstructure of Cement Hydration Products

**DOI:** 10.3390/nano7120429

**Published:** 2017-12-05

**Authors:** Liguo Wang, Shupeng Zhang, Dapeng Zheng, Haibin Yang, Hongzhi Cui, Waiching Tang, Dongxu Li

**Affiliations:** 1 Jiangsu National Synergetic Innovation Center for Advanced Materials (SICAM), Nanjing Tech University, Nanjing 210009, China; wanglg@njtech.edu.cn (L.W.); 815806703@njtech.edu.cn (S.Z.); 2 Guangdong Provincial Key Laboratory of Durability for Marine Civil Engineering, College of Civil Engineering, Shenzhen University, Shenzhen 518060, China; zhengdapeng@student.cumtb.edu.cn (D.Z.); 2150150417@email.szu.edu.cn (H.Y.); 3 School of Architecture and Built Environment, the University of Newcastle, Callaghan, NSW 2308, Australia; patrick.tang@newcastle.edu.au

**Keywords:** graphene oxide (GO), cement hydration products, calcium hydroxide, crystal structure, microstructure

## Abstract

In this study, the effects of graphene oxide (GO) on the microstructure of cement mortars were studied using scanning electron microscopy (SEM), thermogravimetric (TG), and X-ray diffraction (XRD) techniques. Cement mortar samples with different proportions of GO (0.02, 0.04, 0.06, and 0.08 wt % based on the weight of cement) were prepared. The test results showed that GO affected the crystallization of cement hydration products, C–S–H (calcium silicate hydrate is the main hydrate product) and CH (calcium hydroxide). The morphology of hydration products changed with the increase of GO content. Furthermore, the results of XRD analyses showed that the diffraction peak intensity and the crystal grain size of CH (001), (100), (101), and (102) for GO samples increased considerably compared with the control sample. Based on the results, it can be understood that GO can modify the crystal surface of CH, leading to the formation of larger crystals.

## 1. Introduction

Graphene oxide (GO) is one of the graphene derivatives, which basically consists of a several layers of wrinkled two-dimensional carbon sheet with various oxygen-containing functional groups such as hydroxyl, carboxyl, and epoxy groups on its surface and/or between the inter-sheet layers [1]. GO is usually characterized by its excellent properties such as high strength, high toughness and large specific surface area. The active groups not only enlarge the distance between layers of GO sheets [2] but also make them hydrophilic, which allows GO to disperse well in the solution for the formation of intercalation compounds with other materials. Recently, some works have been carried to study the microstructure of graphene reinforced cementitious materials. Lin et al. [3] stated that GO could improve hydration due to its oxygen-containing functional groups. Tong et al. [4] showed that graphene nanoplatelets (GNPs) or graphene oxide nanoplatelets (GONPs) could effectively improve frost resistance of cement-based materials, and could greatly change the microstructure of cement hydration products. The atomic force microscope (AFM) and SEM images showed that the interfacial bond between the GNPs/GONPs and C–S–H gels precipitated around them was well developed. Yang et al. [5] and Li et al. [6] found that GO with a large surface area could be well dispersed in the solution using ultrasonic methods, and consequently the hydration degree and mechanical strength of the cement paste could be enhanced.

In addition to ordinary cement material, several works have also been carried out to study the performances and microstructure of GO reinforced geopolymer and other special cementitious materials. Zhong et al. [7] had prepared GO modified geopolymer nanocomposite for extrusion-based 3D (three dimensional) printing. They realized that some thick water film was formed between GO and aluminosilicate particles resulted in enhancement of complex modulus of the mixture, and led to a better rheology and mechanical performances of geopolymer for 3D printing. In another study, Zhu et al. [8] investigated the influences of GO on hydration progress and microstructure of alkali-activated slag (AAS) cementitious material. They found that increasing GO content could reduce the fluidity of AAS mortar due to the large surface area of GO. They also showed that some larger layered double hydroxides (LDHs) crystal druse with flower-like structure had been formed in the GO modified AAS hydration system. Moreover, in a study by Lu et al. [9], the influences of GO on the performance and microstructure of magnesium potassium phosphate cement (MKPC) were investigated. Similarly, GO reduced the setting time and fluidity of MKPC due to the large surface area. They also reported that GO (0.05 wt %) modified MKPC with denser microstructure showed better mechanical strength, which was attributed to the “filler effect” and good mechanical performances of GO.

However, the influence mechanism of GO on cement hydration products is still uncertain and yet to reveal. This paper mainly studied the effects of GO on morphology and microstructure of cement hydration products. The influence mechanism of GO on cement hydration was revealed using the following test methods: X-ray diffraction (XRD), thermogravimetric (TG), and scanning electron microscopy (SEM) observations. 

## 2. Materials and Methods

### 2.1. Materials and Mix Proportions

#### 2.1.1. Materials

The cement used for this experiment was PII (Portland Type II) 52.5 N ordinary Portland cement supplied by Jiangnan Onoda Cement Co., Ltd. (Nanjing, China). The chemical compositions of Portland cement are shown in Table 1. The siliceous sand used in this study was obtained from China ISO Standard Sand Co., Ltd. (Xiamen, China). Aqueous dispersions of GO (7.4 g/L GO, Laboratory synthesis) and deionized water were used in the experiment.

**Figure 1:**
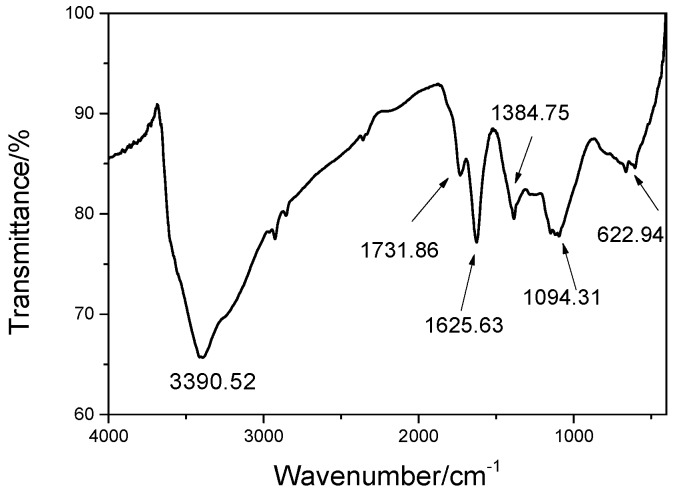
Fourier-transform infrared spectroscopic (FT-IR) spectrum of GO.

**Figure 2:**
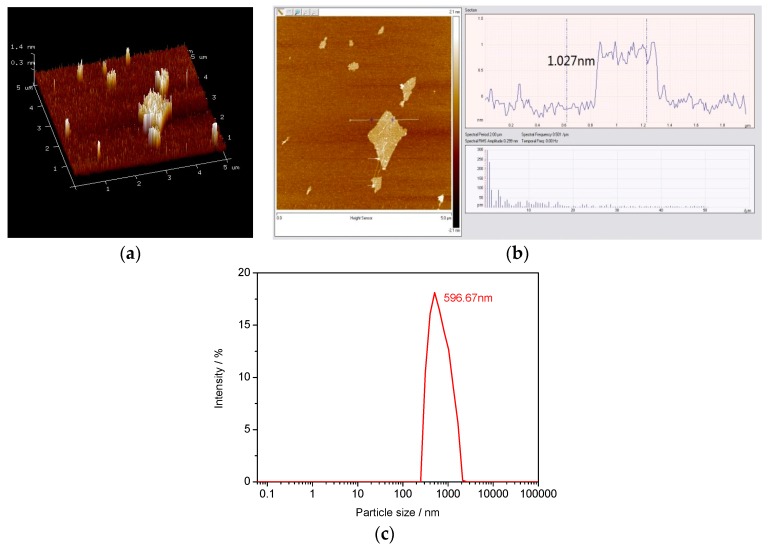
AFM images of the GO and dispersion in solution (**a**) 3D structural characterization of GO nanosheets by AFM test; (**b**) 2D (two dimensional) structural characterization of GO nanosheets by AFM test, (**c**) GO dispersion in solution by dynamic light scattering (DLS).

The Fourier-transform infrared spectroscopic (FT-IR) spectra (Waltham, MA, USA) of GO is shown in Figure 1. The results confirmed that the oxygen functional groups of –OH, –COOH, and –O– were found on the surface of GO, as supported by the new absorption peaks of GO at 3390 cm^−1^ (–OH), 1731 cm^−1^ (–COOH), and 1384 and 1094 cm^−1^ (–O–), respectively [10,11]. These oxygen-containing groups were chemically grafted onto the graphite surface. With the introduction of these additional groups, the interlayer spacing increased and the bonding between the layers were weakened, leading toward the formation of single-layer graphene oxide. The Atomic Force Microscope (AFM) images of the lamellae of GO are displayed in Figure 2a,b. It can be seen from the figures that the size of GO sheet reached the nanometer scale, and the thickness of a single irregular lamella was about 1.027 nm with its length and width at about 600 nm. These results showed that monolayers of graphene oxide could be prepared using the ultrasonic dispersion method, and the GO nanosheets suspension solution was obtained successfully. The effectiveness of the dispersing method as well as the factual particle size distribution of the liquid GO dispersion was obtained by the dynamic light scattering (DLS). The test result is presented in Figure 2c. From Figure 2c, it can be seen that the GO disturbed in a narrow range from 300 nm to 2 µm. The mean size of GO in the solution was 596.67 nm, which is similar with the test result of AFM, therefore, the dispersion of GO in the GO liquid was considered uniform.

#### 2.1.2. Preparation of Cement Mortar with GO

Cement paste samples with different contents of GO (0.02, 0.04, 0.06, and 0.08 wt %) were prepared. Water-to-cement ratio of all samples was 0.40. First, the GO solution was diluted into the deionized water. Then, the mixture was added with polycarboxylate superplasticizer and gently stirred with a glass rod. The superplasticizer used was not only to maintain the workability of cement paste samples, but also acted as a dispersing agent, and the dosage of superplasticizer agent was 0.18 wt %. In the practice, the difference of workability between different samples is small. After that, the mixture solution was agitated using an ultrasonic method (Ultrasonic cleaners, KQ118, power 70 W, Kunshan, China) to achieve homogeneous dispersion of GO in the solution. After 30 min of agitation, the mixture was stirred at a low-speed for 30 s, and cement was subsequently added into the mixture and stirred in a similar manner for another 2 min. When siliceous sand was added, the blend was mixed at a high-speed rotation for 2 min. After mixing, the GO cement samples were immediately poured into a 40 mm × 40 mm × 160 mm mold. The final specimens were striped after 24 h and cured in water at 20 ± 1 °C, until specified ages of testing. Samples were packed in small bottles with anhydrous ethanol for microscopic tests. Microstructural properties of hydrated cubes were evaluated after 28 days of curing. A sample consisted of plain cement mortar without and with 0.8 wt % GO were prepared following the same procedure above.

### 2.2. Testing Procedures

X-ray diffraction (XRD) was used to analyze the hydration products of cement pastes. XRD patterns were recorded at a scanning rate of 2°/min from 2θ = 5° to 2θ = 80° with Cu Kα radiation (λ = 1.5418 Å) on a Bruker D max/RB diffractometer (Billerica, MA, USA). Scanning electron microscope (SEM, JMS-5900, JEOL, Tokyo, Japan) was used to analyze the morphology of cement paste. Differential thermal analysis (TG, NETZSCH, ATA409 NETZSCH, Selb, Germany) was employed to measure the calcium hydroxide content of GO cement samples at different ages (3 and 28 days) through semi-quantitative analysis. In this test, the heating rate was 20 °C/min, and nitrogen was used as a DSC (differential scanning calorimetry) standard purge gas due to its better heat conductivity. The material of the pans was corundum.

## 3. Results and Discussion

### 3.1. Compressive and Flexural Strength of Cement Mortar

**Figure 3:**
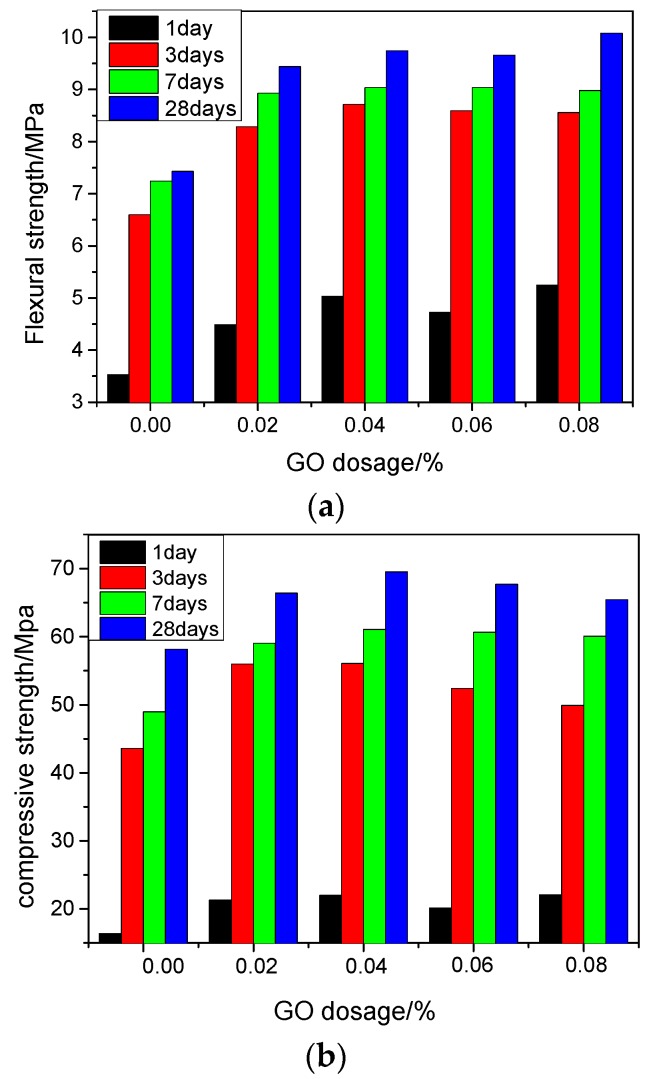
Flexural and compressive strengths of GO cement samples. (**a**) Flexural strength; (**b**) compressive strength.

The effects of GO contents (0, 0.02, 0.04, 0.06, and 0.08 wt %) on flexural and compressive strength of cement paste at different ages are shown in Figure 3. Compared with the control samples, the flexural and compressive strengths of cement paste increased significantly with the addition of GO. The results indicated that GO significantly improved the mechanical properties of cement mortar, with the greater effect on the early age strengths. The effect of GO content on flexural strength improvement is slightly different from what can be observed on compressive strength. When the GO content was 0.02%, the percentage improvement in flexural strength was slightly smaller than that observed in compressive strength at one and three days. At the age of seven days, the flexural strength improvement was greater than that of compressive strength. However the difference became smaller as age increased. When GO content was 0.04%, the 28-day compressive strength increased by 19.5%, whereas the flexural strength increased by 22.8%. When GO content was 0.08%, the 28-day flexural strength improvement was the greatest (27.1%), whereas the compressive strength increased by only 16.4%. Some studies reported that the enhanced mechanism of GO in cement composites is similar to what has been observed in the cement composites with carbon nanotubes (CNTs). It is believed that GO and CNTs can suppress the expansion of crack at the nanometer scale, in other words, GO can play the CNTs’ bridge role in cement paste [12,13] and resulted in a greater improvement in flexural strength. Moreover, GO has a large specific surface area and a two-dimensional corrugated folds of surface, which greatly improved the interface bonding [14,15,16].

### 3.2. Mechanism Analysis of GO Modified Cementitious Material

**Figure 4:**
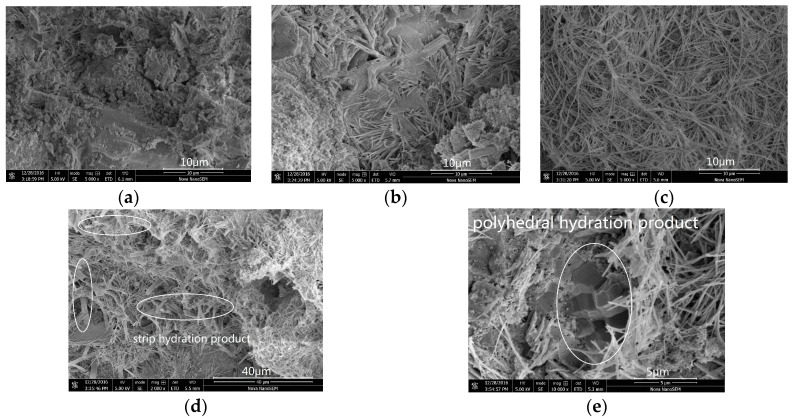
Morphologies of cement hydrates with different GO contents at an age of 28 days. (**a**) Control; (**b**) 0.02% GO; (**c**) 0.04% GO; (**d**) 0.06% GO, many flat and long strip hydration products appeared and embedded in the cement paste; (**e**) 0.08% GO, a polyhedral block shape products appeared and embedded in the cement paste.

Figure 4 shows the SEM micrographs of cement with different GO contents, compared with the plain cement at an age of 28 days. In general, the microstructure of plain cement shown in Figure 4a seemed more compact and accompanied with fewer amounts of needle-like or rod-like hydration products. In contrast, the microstructures of samples with low GO contents (0.02 and 0.04 wt %) have a great amount of needle-like hydration products, as shown in Figure 4b,c. The formation of bridging between the hydration products can also be seen in the above-mentioned figures. When GO content was 0.06 wt %, many flat and long strip hydration products appeared and embedded in the cement paste as shown in Figure 4d. When the GO content was increased to 0.08 wt %, the microstructure exhibited a polyhedral block shape, which was cemented closely with other hydration products as indicated in Figure 4e. These microstructural changes were mainly attributed to the large specific surface area of GO with its high amount of oxygen functional groups, which served as the nucleation sites for the formation of hydration products during the cement hydration process. Apparently, the change in morphology of cement paste was more evident with the increasing content of GO. When the GO content in cement was low, the hydration products were mostly combined together at one end with another, and remained divergent owing to fewer nucleation sites formed. When the GO content increased, the nucleation sites for hydration products also increased and the hydration products tended to form a regular cluster structure to prevent micro-cracks development. Based on these SEM results, it can be shown that GO has the effect of controlling the crystallization and morphology of cement hydration products, which depends on the GO content in cement paste. However, Cui et al. [17] suggested that the chemical compounds of flower-like and polyhedral substances are calcium carbonate crystals, which are the products from carbonation of cementitious hydrates, not from cement hydration.

### 3.3. The Content and Crystallization of Ca(OH)_2_ in Cement Hydration Products

**Figure 5:**
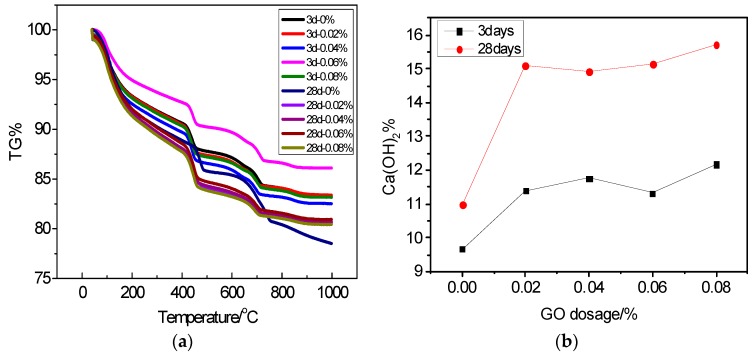
TG curves of cement pastes and mass loss of CH at different ages, (**a**) TG curves of cement paste and (**b**) mass loss of CH.

CH crystals are the second most important hydration products after C–S–H gel [11] as their morphology and particle characteristics play a prominent role on mechanical properties of hardened cement paste. In order to have a further understanding of the influence of GO on cement hydration process, TG was employed to measure the calcium hydroxide content of GO cement paste samples through semi-quantitative analysis. According to the TG curves of cement pastes, shown in Figure 5a, the mass loss of CH at different ages was measured and found between 400 and 500 °C [18,19]. It can be seen from Figure 5b that the content of CH in GO samples was always higher than that of the control sample, and tended to increase with increasing GO content. At an age of three days, the contents of CH in cement pastes with 0, 0.02, 0.04, 0.06, and 0.08 wt %. GO were 9.0, 12.5, 8.2, and 16.5 wt % higher than the control sample, respectively. It is worth noting that the cement hydration products in cement paste is very complex and the CH content is not stable in early stage of cement hydration. Normally, the CH content in the cement pastes with different GO dosages tends to stabilize as the cement hydration time increases. At 28 days, the contents of CH in the cement pastes were increased by 36.8, 35.3, 37.2, and 42.4 wt %, accordingly. It has been reported that the CH content is strongly related with the cement paste hydration degree [20]. Therefore, according to the present results, it can be stated that the presence of GO in cement can promote the hydration of cement paste at different hydration stages. Kai [21] also reported the similar experimental results. It has been generally accepted that the hydration process of cement is mainly controlled by the nucleation and growth of cement hydration products. Thus, it can be stated that the effect of GO on cement nucleation also plays a significant role in hydration degree of GO reinforced cement.

**Figure 6:**
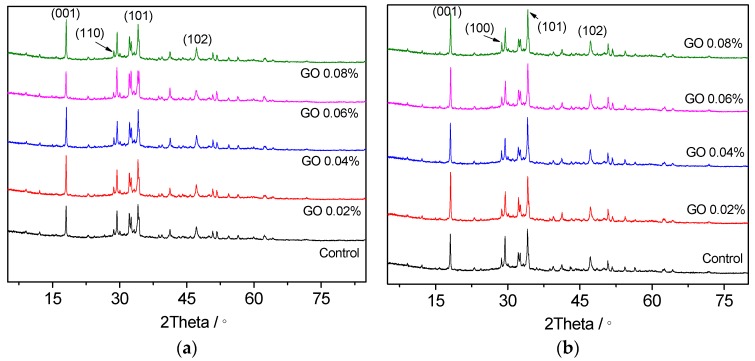
XRD patterns of hydration products. The CH crystal peaks were shown in both plots. (**a**) 3 days; (**b**) 28 days.

It is commonly known that CH crystals clearly present a hexagonal plate shape in hardened cement paste [22]. If the shape of CH can be monitored to become a rod-like or needle-like appearance in cement paste, the mechanical properties of cement-based materials can be strengthened [23]. The effect of different GO contents on formation of CH crystals in cement paste was measured by XRD and the results are shown in Figure 6. The diffraction peaks of CH in the (001), (100), (101), and (102) planes were 18.1°, 28.7°, 34.1°, and 47.1° respectively. Furthermore, the sizes of CH crystals were calculated using the Debye-Scherrer equation as expressed in Equation (1) [24].

(1) $$D=\frac{K\lambda }{\beta \cos\theta }$$

where *D* is the size of grains in the direction perpendicular to the crystal plane; *λ* is the X-ray wavelength; *θ* is the diffraction angle; *β* is the diffraction peak half width; K is a constant equal to 0.89.

Table 2 and Table 3 show the results of diffraction peak intensity and the CH crystal size of GO samples after 3 and 28 days of curing. It can be seen from the tables that the GO content showed insignificant influence on the diffraction angle of CH, as the unit cell parameters of CH remained unchanged. Therefore, only the differences in CH crystallization between cement paste with and without GO were compared and discussed. At three days, the diffraction peak intensity of the (001) plane for control sample was 1517, and the corresponding crystal size was 57.2 nm. On the other hand, the diffraction peak intensities of the (001) plane for GO samples were 25.8, 27.1, 17.9, and 27.1 wt % larger than that of the control sample when 0.02, 0.04, 0.06, and 0.08 wt % GO content were used. The size of CH crystals was 16.1, 7.3, 9.6, and 13.5 wt % higher than that of the control sample. At 28 days, the diffraction peak intensity of the (001) plane for control sample was 1725, and the corresponding crystal size was 59.2 nm. When the curing age increased, the crystallization of CH in the control sample increased, and the crystal size became larger. For GO samples at 28 days, their diffraction peak intensities of the (001) plane were 30.6, 8.2, 11.4, and 12.7 wt % higher than that of the control sample when the GO was added. For the three days samples with the same content of GO, the corresponding sizes of crystal were 14.2, 7.1, 13.5, and 13.3 wt % bigger than the control sample, respectively. The (100), (101), and (102) planes showed a similar trend with the increase of GO content. The results indicate that the change in diffraction peak intensity and crystal grain size was mainly affected by the GO content in cement paste. Therefore, it can be stated that GO can modify the crystal surface of CH, leading to the formation of larger crystal sizes that promote crystallization of CH in the cement paste.

## 4. Conclusions and Recommendations

GO significantly improved the mechanical properties of cement mortar, with a greater effect on the early age strengths. In general, the flexural and compressive strengths of cement paste increased significantly with the increase of GO content. When 0.08% GO was added, the 28-day flexural strength improvement was the greatest (27.1%), whereas the compressive strength increased by 16.4%. The morphology of the hydration products changed with the increase of GO content. The microstructural change was mainly attributed to the large specific surface area of GO accompanied with a high amount of oxygen functional groups, which served as nucleation sites in the formation of hydration products during the cement hydration process. The XRD results indicated that GO could promote the formation of CH crystals. The diffraction intensity and crystal size of CH in their different planes observed in GO cement samples were greater compared with the control sample. So, it can be concluded that GO could lead to the formation of larger CH crystal sizes and promote higher CH crystallizations in the cement paste. 

Properties of GO modified cement, such as fracture, porosity, and durability have not been fully investigated, but it would be worthwhile to carry them out in the near future.

## Figures and Tables

**Table 1:** Chemical compositions of Portland cement (wt %).

Type	CaO	SiO_2_	Al_2_O_3_	Fe_2_O_3_	SO_3_	MgO	K_2_O	Ignition Loss
PII 52.5 N	64.95	18.31	4.21	2.95	4.22	0.64	0.788	3.21

**Table 2:** CH diffraction peak intensity and crystal size at 3 days.

hkl	GO (%)	2θ (°)	Peak Height	Crystal Size (nm)	hkl	GO (%)	2θ (°)	Peak Height	Crystal Size (nm)
001	0	18.021	1517	57.2	101	0	34.080	1711	21.4
	0.02	18.021	1909	66.4		0.02	34.080	1907	28.6
	0.04	18.080	1928	61.4		0.04	34.121	1975	29.4
	0.06	18.019	1789	62.7		0.06	34.061	1852	31.0
	0.08	18.059	1929	64.9		0.08	34.100	1861	26.4
100	0	28.660	368	51.4	102	0	47.100	647	24.4
	0.02	28.679	411	56.3		0.02	47.099	711	29.2
	0.04	28.719	407	68.4		0.04	47.159	718	27
	0.06	28.659	404	55.4		0.06	47.080	657	26
	0.08	28.699	371	64		0.08	47.120	677	26.3

**Table 3:** CH diffraction peak intensity and crystal size at 28 days.

hkl	GO (%)	2θ (°)	Peak Height	Crystal Size (nm)	hkl	GO (%)	2θ (°)	Peak Height	Crystal Size (nm)
001	0	18.039	1725	59.2	101	0	34.099	2040	29.7
	0.02	18.098	2254	67.6		0.02	34.140	2211	32.8
	0.04	18.059	1867	63.4		0.04	34.101	2281	40.9
	0.06	18.100	1922	67.2		0.06	34.141	2301	34.9
	0.08	18.100	1944	67.1		0.08	34.141	2287	34.4
100	0	28.68	509	60.9	102	0	47.120	766	28.9
	0.02	28.739	518	47.8		0.02	47.160	775	27.6
	0.04	28.698	532	67.9		0.04	47.121	802	29.9
	0.06	28.740	533	49.2		0.06	47.180	806	29.7
	0.08	28.739	525	61.3		0.08	47.161	789	30.4
